# Perceptions and behavior of clinical researchers and research support staff regarding data FAIRification

**DOI:** 10.1038/s41597-022-01325-2

**Published:** 2022-05-27

**Authors:** Martijn G. Kersloot, Ameen Abu-Hanna, Ronald Cornet, Derk L. Arts

**Affiliations:** 1 Amsterdam UMC location University of Amsterdam, Department of Medical Informatics, Amsterdam, Meibergdreef 9, The Netherlands; 2 Castor EDC, Amsterdam, The Netherlands; 3 Amsterdam Public Health, Methodology, Amsterdam, The Netherlands

**Keywords:** Research data, Medical research

## Abstract

The FAIR Data Principles are being rapidly adopted by many research institutes and funders worldwide. This study aimed to assess the awareness and attitudes of clinical researchers and research support staff regarding data FAIRification. A questionnaire was distributed to researchers and support staff in six Dutch University Medical Centers and Electronic Data Capture platform users. 164 researchers and 21 support staff members completed the questionnaire. 62.8% of the researchers and 81.0% of the support staff are currently undertaking at least some effort to achieve any aspect of FAIR, 11.0% and 23.8%, respectively, address all aspects. Only 46.6% of the researchers add metadata to their datasets, 39.7% add metadata to data elements, and 35.9% deposit their data in a repository. 94.7% of the researchers are aware of the usefulness of their data being FAIR for others and 89.3% are, given the right resources and support, willing to FAIRify their data. Institutions and funders should, therefore, develop FAIRification training and tools and should (financially) support researchers and staff throughout the process.

## Introduction

Since their publication in 2016, the FAIR Data Principles are being rapidly adopted by many research institutes and funders^1^. The principles state that data should be FAIR, both for humans and machines^2^, and the need to organize and manage data according to the principles is becoming more important in the data-driven landscape of medicine^3^. There are many ongoing efforts that directly or indirectly contribute to the objective of making FAIR Data a reality^4^.

Researchers are increasingly asked or obliged to make their data (more) FAIR, either by funders (e.g. Horizon Europe^5^ and The Dutch Research Council (NWO)^6^) or their institutions (e.g., the Leiden University Medical Center^7^ and Radboud University^8^). Research institutes often support individual researchers in the FAIRification of their data by offering help from trained research support staff (e.g., data stewards) that assist them during the FAIRification process^3^. However, since FAIR is not a standard and the principles do not offer explicit guidance on the methodologies that should be used for data FAIRification, there are many different approaches to FAIRify data (i.e., make data FAIR)^9,10^. Various workflows have been published to guide researchers and researcher support staff during the FAIRification process^11–13^. For the majority of the steps in these workflows, different types of expertise are required, and these steps should, therefore, be carried out in a multidisciplinary team guided by FAIR data steward(s), instead of by individual researchers or research support staff^12,14^. We, therefore, identify a gap between the expectations of funders and institutions and researchers’ and research support staff’s abilities to make data FAIR.

In order to determine what resources are needed to bridge this gap, more information about the knowledge and perceptions of researchers and research support staff (staff supporting researchers with data management and data stewardship tasks and questions, e.g., data stewards and data managers) regarding data FAIRification is needed. To our knowledge, this information has not been collected before. The aim of this study was, therefore, to gain insight into the current efforts of researchers and research support staff to share data and to make the data they collect more FAIR, through an online questionnaire. In addition, this study focuses on the perceptions of researchers regarding data FAIRification and their familiarity with their organization’s Research Data Management policy.

## Methods

### Development and testing of the questionnaire

To determine the perceptions and current efforts of researchers and research support staff, an online English questionnaire was developed. The questionnaire consisted of six parts: 1) Demographics, 2) Familiarity with data sharing, 3) Familiarity with the organization’s research data management policy, 4) Familiarity with the FAIR Data Principles, 5) Effort spent in making data FAIR, and 6) Statements regarding FAIR Data. Five stakeholders involved in clinical research from different institutes (two PhD candidates, one post-doctoral researcher, one data management consultant, and one data steward) tested the questionnaire and we modified the questionnaire based on their feedback.

#### Effort spent in making data FAIR

Researchers and research support staff were asked to report their current efforts in making data Findable, Accessible, Interoperable, and Reusable. Since there is no established quantitative measure available for self-reporting FAIRification efforts, we used a four-point Likert scale for every aspect of FAIR (no effort, very little effort, some effort, a lot of effort). Box 1 lists the descriptions of the aspects of FAIR that were used throughout the questionnaire.

Box 1 Description of the aspects of FAIR used in the questionnaire, based on^2^.

**Findable**Data should be easy to find by both humans and computer systems based on a description of the dataset (metadata).

**Accessible**Data should be stored for the long term such that they can be easily accessed with well-defined license and access conditions. This does not necessarily mean open access.

**Interoperable**Data should be ready to be combined with other datasets by humans as well as computer systems.

**Reusable**Data should be ready to be used for future research and to be processed further using computational methods.

#### Statements regarding FAIR data

**Fig. 1:**
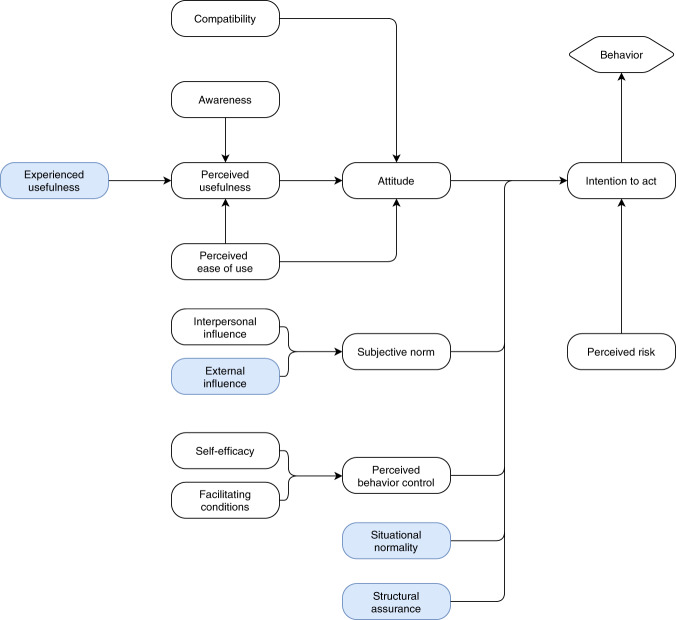
Adopted part of the model described in^15^ that focuses on working processes and human attitudes. Adapted parts are highlighted.

Since data FAIRification is largely a standardization process, where one makes sure that data are exposed in a standardized manner and accompanied by structured and standardized metadata, we used an adapted version of Joukes *et al*.’s model^15^ for the last part of the questionnaire. Joukes *et al*.’s model measures the perceived level of adoption of structured and standardized recording among healthcare professionals and is based on two models that use the TAM as underlying model, which aims to explain how users come to accept and use a technology. The model was used to both gain insight into the perceptions of researchers and research support staff regarding data FAIRification (or technology in the TAM) and to predict how these perceptions influence the behavior of researchers. We adopted the part of the model that focuses on working processes and human attitudes (Fig. 1). *Experienced usefulness* was added as an additional factor for *Perceived usefulness*, for if FAIR Data aided in previous research projects, it might influence the perceived usefulness. Additionally, *Governmental influence* was replaced by *External influence*, for there are more organizations that can influence the *Subjective norm* of a researcher or supporting staff (e.g., Funders). *Institutional trust* was removed from the model, since we hypothesized that *Situational normality* and *Structural assurance* for data FAIRification are not affiliated with the trust of researchers and staff in their institution. Descriptions of each factor in the model are listed in Table 1. Each question was a statement that could be answered on a five-point Likert scale (strongly disagree (1), disagree (2), neutral (3), agree (4), strongly agree (5)). All statements and their associated factors are listed in Supplementary Table 1.

**Table 1:** Factors originating from the model and their descriptions.

Model concepts	Explanation
Attitude	What the researcher thinks of FAIR Data
Awareness	Whether the researcher knows that it is important that their data should be made FAIR
Behavior	A number of facets that indicate whether the researcher is already making their research data FAIR
Compatibility	Whether FAIRification of research data fits the work processes of the researcher
Experienced usefulness	Whether FAIR Data aided in the researcher’s research projects
External influence	Whether the external organizations (i.e., funders) promotes the FAIRification of research data
Facilitating conditions	Whether there is enough time and there are appropriate tools available to make research data FAIR
Intention to act	Whether the researcher wants to make their research data FAIR
Interpersonal influence	Whether the supervisor or organization promotes the FAIRification of research data
Perceived behavioral control	Whether it is within the researcher’s control to make data FAIR
Perceived ease of use	The overall opinion of the researcher on the usability of the FAIRification process
Perceived risk	Whether the reuse of data can harm the patients’ privacy and or safety
Perceived usefulness	Whether FAIR Data aids in the researcher’s research projects
Self-efficacy	Whether the researcher is capable of making their research data FAIR
Situational normality	Whether it is normal in the organization to make data FAIR
Structural assurance	Whether the organization ensures that there is a policy on FAIR Data stewardship
Subjective norm	Whether the researcher makes their research data FAIR because colleagues expect this

### Participants and data collection

Participants were recruited through an invitation in a popup in EDC platform Castor EDC^16^ and by email in six out of the seven Dutch University Medical Centers. We used a generic invitation text focusing on Research Data Management instead of FAIR Data, in order to ensure that potential participants that did not know about FAIR were also included. Researchers and staff were eligible to participate in our study if they: 1) set up databases for clinical research (e.g., PhD candidates), or 2) support clinical researchers with the creation of these databases (e.g., data stewards). Users of the EDC platform that have previously built a database in the system, among which researchers and research support staff, received a popup after logging in to the system asking them to participate in the study. Research data desk coordinators of five University Medical Centers sent out invitation emails to a random group of researchers and research support staff in their center. PhD student associations of three University Medical Centers sent out invitation emails to their members. In order to enhance the response rate, a lottery-based incentive of ten 50 gift cards was offered. Data was collected in Castor EDC^16^ between November 27, 2020, and February 27, 2021.

### Ethical aspects

The study design was submitted to the Medical Ethics Review Committee of the Academic Medical Center in Amsterdam, the Netherlands, and was exempt from review (reference W20_499#20.550). Consent of respondents was required before the questionnaire could be opened.

### Data analysis

Statistical analyses were performed using R (version 4.0.2)^17^. Questionnaires with complete answers to mandatory questions were included in the analysis. A p-value below 0.05 was considered significant. The p-values were adjusted for multiple testing using Bonferroni correction.

#### Perceptions of researchers and research support staff

The scores per factor of researchers with knowledge of FAIR and researchers without knowledge of FAIR were compared with the Mann–Whitney U test.

#### Behavior of researchers

Similar to Joukes *et al*.^15^, we performed SEM using the PLS method. PLS is a causal-predictive approach to SEM and is designed to provide causal explanations^18^. We used PLS because it is designed to provide causal explanations and predictions^18^ and works efficiently with small sample sizes^19^. In PLS terminology, the statements about FAIR Data in our questionnaire are the observed variables that are linked to the latent variables, which are the factors in our model. The plspm R package^20^ (version 0.4.9) was used for the analysis. Data from researchers was used, for researchers are responsible for (FAIR) data management throughout their research project, while support staff only assist them in this process.

In order to determine the performance of the model we used the performance measures and their target values described by Hair *et al*.^21,22^. Indicator reliability was derived from the outer loadings of the observed variables. A loading > 0.708 indicates that the observed variable explains more than 50% of the latent variable’s variance and is, therefore, recommended. Discriminant validity was assessed using Henseler *et al*.’s HTMT ratio^23^, where an HTMT ≤ 0.90 (or ≤ 0.85 if the latent variables are conceptually more distinct) indicates discriminant validity. Internal consistency reliability was calculated using Dillon Goldstein’s rho, where a value > 0.60 is considered acceptable in exploratory research. The convergent validity of each latent variable was calculated using the AVE. An acceptable AVE is ≥ 0.50, which indicates that the latent variable explains at least 50% of the variance of its items.

## Results

**Fig. 2:**
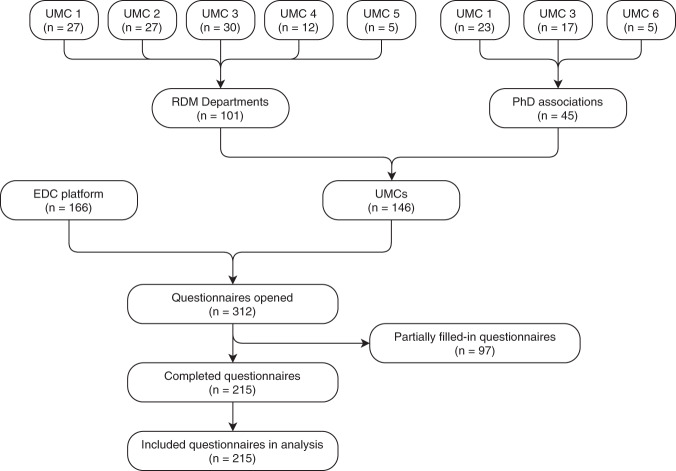
Flow diagram of participants.

In total, 312 respondents opened the questionnaire. 215 respondents completed the questionnaire and were included in the analysis (Fig. 2). Of these, 76.3% were conducting research themselves and 9.8% had a supporting function (Table 2). The remaining 14% had another profession (e.g., doctors and nurses). The majority of the respondents were working in an academic hospital (84.2%). Of all respondents, 60.5% had heard of the FAIR Data Principles and 44.7% reported that they knew their definition.

**Table 2:** Demographics of the included respondents grouped by self-reported knowledge of the FAIR Data Principles.

	All respondents (N = 215)		FAIR knowledge (N = 96)		No FAIR knowledge (N = 119)	
	n	(%)	n	(%)	n	(%)
Profession						
Researcher	164	(76.3)	74	(77.1)	90	(75.6)
PhD candidate	139	(64.7)	59	(61.5)	80	(67.2)
Assistant professor	4	(1.86)	2	(2.08)	2	(1.68)
Professor	3	(1.40)	2	(2.08)	1	(0.84)
Other	5	(2.33)	2	(2.08)	3	(2.52)
Post-doc	11	(5.12)	8	(8.33)	3	(2.52)
Associate professor	2	(0.93)	1	(1.04)	1	(0.84)
Support staff	21	(9.77)	16	(16.7)	5	(4.20)
Data Steward	10	(4.65)	8	(8.33)	2	(1.68)
Data Manager	6	(2.79)	5	(5.21)	1	(0.84)
Other	4	(1.86)	3	(3.12)	1	(0.84)
Other	30	(14.0)	6	(6.25)	24	(20.2)
Age						
<30	130	(60.5)	49	(51.0)	81	(68.1)
30–39	51	(23.7)	28	(29.2)	23	(19.3)
40–49	18	(8.37)	8	(8.33)	10	(8.40)
50–59	13	(6.05)	10	(10.42)	3	(2.52)
≥60	2	(0.93)	0	(0.00)	2	(1.68)
Rather not tell	1	(0.47)	1	(1.04)	0	(0.00)
Primary Institution						
Academic hospital	181	(84.2)	85	(88.5)	96	(80.7)
University	11	(5.12)	3	(3.12)	8	(6.72)
Teaching hospital	12	(5.58)	3	(3.12)	9	(7.56)
Hospital	6	(2.79)	2	(2.08)	4	(3.36)
Contract research organization	2	(0.93)	0	(0.00)	2	(1.68)
Other	3	(1.40)	3	(3.12)	0	(0.00)
Research experience						
<1 year	28	(13.0)	8	(8.33)	20	(16.8)
1–2 years	48	(22.3)	22	(22.9)	26	(21.8)
2–4 years	62	(28.8)	20	(20.8)	42	(35.3)
4–6 years	32	(14.9)	15	(15.6)	17	(14.3)
≥6 years	45	(20.9)	31	(32.3)	14	(11.8)

### Data sharing

Of all researchers, 79.5% stated that they have shared “raw” research data with others before (Table 3). The most common ways to share data were via a shared network drive in the researcher’s institution (56.2%) and via email (43.8%). 35.9% of the researchers that have shared data, shared data via data repositories (28.1% and 11.7% for an internal and external data repository respectively). 14.1% of the researchers that share data have published their data as a scientific publication, either as stand-alone data publication (2.34%) or appendix or supplementary information appended to a publication (14.1%). Other sharing methods that researchers mentioned in free text include a secure file transfer service (4.69%), an Electronic Data Capture system (1.56%), FTP transfer (0.78%), and an institution’s Digital Research Environment (0.78%).

**Table 3:** Current data sharing approaches of researchers.

	Researchers (N = 164)	
	n	(%)
Shares research data	128	(79.5)
**Data sharing methods (N = 128)**		
Shared network drive	72	(56.2)
Email	56	(43.8)
Data repository of organization	36	(28.1)
USB Flash drive	23	(18.0)
Appendix/supplementary information in a scientific publication	18	(14.1)
External data repository	15	(11.7)
Cloud storage	18	(14.1)
Stand-alone data publication in a data journal	3	(2.34)
Other	12	(9.38)
**Data sharing with (N = 128)**		
Researchers working on the same research project	118	(92.2)
Researchers not working on the same research project, personally known	16	(12.5)
Researchers not working on the same research project, not personally known	10	(7.81)
Research project partners and funders	13	(10.2)
Other	4	(3.12)

92.2% of the researchers that share data do so with coworkers that are working on the same research project. 18.0% of the researchers that share data do so with others that are not working on the same research project, both known and not known by the researcher (12.5% and 7.81% respectively). Other recipients of the data include (reviewers of) scientific journals (1.56%) and students that are supervised by the researcher (0.78%).

### Research Data Management policy

A majority of the researchers (85.4%) and support staff (90.5%) stated that their institution had a policy regarding Research Data Management (Table 4). 1.83% of the researchers stated that their institution had no such policy. 12.8% of the researchers and 9.52% of the support staff were not aware of such a policy or were unsure. 86.4% of the researchers that were aware of their institution’s policy knew whom to contact if they had questions regarding this policy. For policy-aware research support staff, 100% knew whom to contact in case of questions. 55.7% of the researchers indicated that FAIR Data was incorporated in this policy, 2.86% indicated that it was not part of the policy and 41.4% did not know if FAIR was part of the policy. For research support staff, this was 73.7%, 5.26%, and 21.1% respectively.

**Table 4:** Researchers’ and research support staff’s awareness of their institute’s Research Data Management (RDM) policy.

	Researchers (N = 164)		Support staff (N = 21)	
	n	(%)	n	(%)
Organization has policy	140	(85.4)	19	(90.5)
Organization does not have policy	3	(1.83)	0	(0.00)
Not aware of this policy or not sure	21	(12.8)	2	(9.52)
**Who to contact regarding RDM policy**^†^				
A specific person	40	(28.6)	3	(15.8)
A specific department	81	(57.9)	16	(84.2)
Does not know	19	(13.6)	0	(0.00)
**FAIR Data**^†^				
Is part of policy	78	(55.7)	14	(73.7)
Is not part of policy	4	(2.86)	1	(5.26)
Does not know	58	(41.4)	4	(21.1)

^†^For researchers and support staff aware of their organization’s policy, N = 140 and N = 19 respectively.

### Effort spent in making research data FAIR

**Fig. 3:**
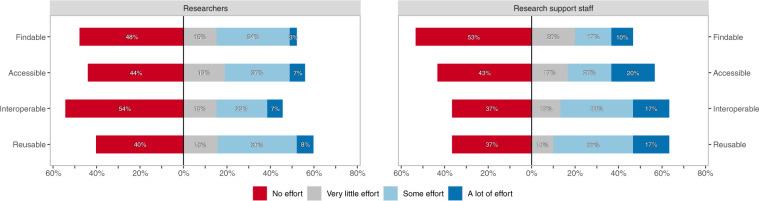
Researchers’ and research support staff’s effort spent in making research data FAIR, per FAIR aspect.

All respondents were given a short explanation of the FAIR Data Principles and each aspect of FAIR. After reading this explanation, 81.1% of the researchers and 81.0% of the research support staff reported that they have spent effort (very little to a lot) to make their research data – or the research data they work with – more Findable, Accessible, Interoperable, or Reusable (*any* aspect of FAIR). Regarding *all* aspects of FAIR, this was 25.6% and 33.3%, respectively. 62.8% of the researchers and 81.0% of the research support staff achieved *any* aspect of FAIR with some or a lot of effort. For *all* aspects this was 11.0% and 23.8%, respectively. Figure 3 shows the effort spent per aspect of FAIR of both groups. Of all researchers, 51.8% spent effort to make their data more Findable, 57.3% spent effort to make their data more Accessible, 47.0% spent effort to make their data more Interoperable, and 60.4% spent effort to make their data more Reusable. For research support staff this was 52.4%, 57.1%, 71.4%, and 66.7% respectively.

### Perceptions of researchers and research support staff

Table 5 lists the scores per latent variable (factor in the model) for researchers and research support staff. In six cases there was a significant difference in score between researchers with knowledge of the FAIR Data Principles and researchers without this knowledge (Awareness, Compatibility, External influence, Facilitating conditions, Interpersonal influence, Structural assurance). Research support staff had higher scores for all factors compared to all researchers, with the exception of Experienced usefulness and Perceived risk. Supplementary Table 2 lists the mean scores per observed variable (question in the questionnaire) for researchers and research support staff. The distribution of the scores can be found in Supplementary Table 3.

**Table 5:** Factors from the model (latent variables) and their scores for researchers and researcher support staff.

Variable	Researchers							Support staff	
	All (N = 164)		FAIR knowledge (N = 74)		No FAIR knowledge (N = 90)		P-value	All (N = 21)	
	Mean	SD	Mean	SD	Mean	SD		Mean	SD
Attitude	4.11	0.52	4.13	0.61	4.09	0.47	>0.999	4.12	0.73
Awareness	3.64	0.59	3.89	0.58	3.51	0.60	0.002**	4.15	0.69
Behavior	2.90	0.69	3.10	0.63	2.77	0.73	0.053	3.19	0.77
Compatibility	3.25	0.95	3.59	0.81	3.04	1.04	0.003**	3.80	1.01
Experienced usefulness^†^	2.98	1.03	2.92	1.10	2.93	1.14	>0.999	2.48	1.62
External influence	2.86	0.99	3.21	0.96	2.65	0.96	0.004**	3.42	0.98
Facilitating conditions	2.73	0.72	3.04	0.73	2.58	0.73	<0.001***	3.44	0.85
Intention to act	3.21	0.71	3.28	0.74	3.18	0.66	>0.999	3.35	0.59
Interpersonal influence	2.71	0.87	3.04	0.82	2.51	0.85	<0.001***	3.26	0.75
Perceived behavioral control	2.96	0.96	3.12	1.00	2.89	0.87	>0.999	3.35	0.75
Perceived ease of use	3.02	0.97	3.18	0.95	2.93	1.01	>0.999	3.25	1.12
Perceived risk	2.44	0.76	2.43	0.89	2.44	0.72	>0.999	2.40	1.15
Perceived usefulness	3.78	0.61	3.81	0.70	3.79	0.58	>0.999	4.00	0.82
Self-efficacy	3.45	0.55	3.57	0.48	3.38	0.61	0.681	3.67	0.57
Situational normality	3.01	0.92	3.26	0.87	2.83	0.92	0.053	3.30	0.86
Structural assurance	3.10	0.82	3.52	0.71	2.84	0.80	<0.001***	3.79	0.61
Subjective norm	2.57	0.98	2.73	0.99	2.52	0.95	>0.999	3.10	0.79

Scores on a five-point rating scale (strongly disagree (1), disagree (2), neutral (3), agree (4), strongly agree (5)).

*p < 0.05, **p < 0.001, ***p < 0.001.

^†^Only applies if the researcher (all, with FAIR knowledge, and without FAIR knowledge) or research support staff member has spent effort to make data more FAIR. N = 133, N = 61, N = 72, N = 17, respectively.

#### Attitude of researchers

Of all researchers, 94.7% stated that their research data being FAIR would be useful for others and 73.3% stated that it would be useful for themselves. With regard to other people’s research data being FAIR, 90.1% of the researchers found this useful for others and 85.5% of the researchers found this useful for themselves.

#### Self-efficacy of researchers

**Fig. 4:**
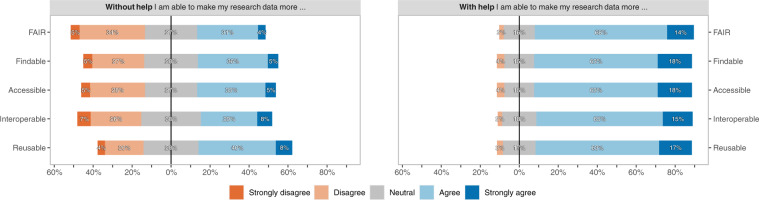
Self-efficacy of researchers in making research data FAIR without and with help, per FAIR aspect.

Figure 4 shows the self-reported self-efficacy of the researchers in making data FAIR. 35.1% of the researchers state that they are able to make their data more Findable, Accessible, Interoperable, and Reusable by themselves. Regarding the individual aspects, F, A, I, and R, this is 41.2%, 40.4%, 36.6%, and 48.1%, respectively. 81.6% of the researchers state that they are able to make their data more FAIR when they receive help. For the individual FAIR aspects this is 81.0%, 81.0%, 80.2%, and 80.2%, respectively.

#### Intention to act of researchers

89.3% of all researchers state that they would intend to make their research data FAIR when they have the possibility and resources to do so. 59.5% would still make their research data FAIR when they have to invest an extra amount of their own time and 27.5% would still do so when they have to spend an extra amount of their budget. If researchers would have to spend both an extra amount of their time and budget, 24.4% of them stated that they would still intend to make their data FAIR.

### Behavior of researchers

In six of the 12 latent variables with more than one observed variable (Attitude, Experienced Usefulness, External Influence, Intention to Act, Interpersonal Influence, Structural Assurance), all loadings of the observed variables (Supplementary Table 4) were above Hair *et al*.’s limit of 0.708^21^ and indicate acceptable item reliability. Four of the latent variables (Awareness, Facilitating Conditions, Perceived Risk, Perceived Usefulness) contained observed variables that were both above and below the limit. All latent variables had a Dillon Goldstein’s rho > 0.787 (Supplementary Table 5), and were above the limit of 0.6 suggested by Hair *et al*^21^, indicating acceptable consistency reliability for exploratory research. Seven of the 12 variables (Attitude, Experienced Usefulness, External Influence, Intention to act, Interpersonal influence, Perceived risk, Structural assurance) had an AVE > 0.5 (Supplementary Table 6), which indicates that these latent variables explain at least 50% of the variance of the associated observed variables. All latent variables had a HTMT ratio ≤ 0.85 (Supplementary Table 7), indicating that there are no discriminant validity problems. Researcher’s awareness of whom to contact or where to find help regarding data FAIRification (Awareness and Facilitating conditions) have higher loadings on the latent variable Structural assurance. Being able to make data more FAIR (in general), Findable, Accessible, and Interoperable by yourself (Self-efficacy) have higher loadings on the latent variable Perceived Ease of Use. Lastly, being able to make data more Reusable by yourself (Self-efficacy) has a higher loading on the latent variable Compatibility.

**Fig. 5:**
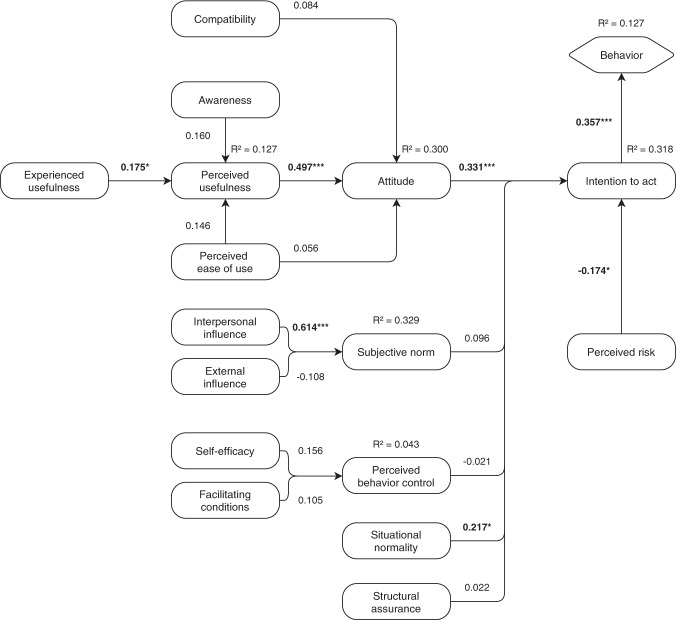
The (structural) model with path coefficients and coefficients of determination (R^2^) for researchers. *p < 0.05, **p < 0.001, ***p < 0.001.

Figure 5 shows the model with path coefficients and coefficients of determination. Path coefficients indicate the effect of a latent variable that is assumed to be a cause on another variable. The coefficients of determination explain the strength of the linear relationship between two latent variables. Seven out of 17 path coefficients were significant. The coefficients of determination (R^2^) ranged from 0.043 (Perceived behavior control) to 0.329 (Subjective norm).

## Discussion

To gain insight into clinical researchers’ and research support staff’s knowledge and perceptions of data FAIRification and experience with making data FAIR, we conducted an online questionnaire. We found that 60.5% of the respondents had heard of the FAIR Data Principles and, after explaining the FAIR Data Principles to the respondents, 62.8% of the researchers and 81.0% of the support staff have spent at least some effort achieving any aspect of FAIR for their data. For all aspects of FAIR this was 11.0% and 23.8%, respectively. Nearly all researchers (94.5%) found the FAIRification of their data useful for others and 89.3% would like to make their data FAIR when they have the opportunity and resources. However, the majority of the researchers (81.6%) stated that they need help to do so.

### FAIRification efforts

A remarkable amount of researchers (81.1%) and support staff (81.0%) stated that they have spent very little or more effort into achieving *any* aspect of FAIR for their research data. However, we believe that *very little effort* spent in the FAIRification of data should be interpreted as *no effort*, for one may be more inclined to say that they have at least spent a bit of effort in making data FAIR, instead of no effort at all. When very little FAIRification efforts are neglected, we see that only a minority of the researchers (11.0%) and support staff (33.3%) spent ‘real’ effort on achieving *all* the different aspects of FAIR. Regarding these aspects, most researchers spent their efforts on improving the data’s 1) Reusability, followed by 2) Findability and Accessibility (equal), and 3) Interoperability. For research support staff this is 1) Reusability, 2) Interoperability, 3) Accessibility, and 4) Findability. Since Reusability is a result of the other three aspects, it is logical that most effort is spent on that aspect. The effort spent on Interoperability is coherent with the fact that it is common that support staff assist researchers in making data more Interoperable.

### Data sharing

Most researchers have shared data before, but only a minority (18.0%) shared data with others not working on the same research project. Moreover, researchers prefer sharing data via shared network drives and email over the use of data repositories and data publications, which hampers data findability and introduces security risks. In addition, only a minority of the researchers stated that they have spent some effort in increasing the findability of their data and a minority stated that they add metadata to their datasets and data elements. To achieve a FAIR Data ecosystem, data should be accompanied by metadata, and both the data and metadata should be findable in both a human- and machine-readable manner^2^. In the cases where the data cannot be shared directly, which might often be the case in medical research due to the sensitive nature of health data, the sharing of metadata can already be useful. Furthermore, de-identified and anonymized datasets that aim to preserve the patient’s privacy can be used for sharing, or data sharing agreements can be made to ensure the data is handled safely^24^. Research institutes should offer support and the technical infrastructure for researchers to make their metadata or de-identified and anonymized datasets available, as well as to support them in the drafting of the aforementioned agreements.

### Knowledge of the FAIR Data principles

A minority of the researchers and majority of the research support staff stated that they knew the definition of the FAIR Data Principles. Structural assurances by institutes that support the process of making data FAIR presumably lead to a better understanding of the FAIR Data Principles among personnel, for researchers that knew the definition of FAIR stated that their institution had these assurances in place. Researchers that knew the definition of the FAIR Data Principles were more aware of their importance. Lastly, these researchers felt that the process of making data FAIR was aligned with their work processes and were more inclined to make their data FAIR. To increase the adoption of the FAIR Data Principles, we, therefore, believe that institutions should offer structural support for researchers. This could include implementing a policy on making data FAIR and providing researchers with infrastructure to store FAIRified data, but also appointing FAIR Data stewards and providing training on data FAIRification.

### Research Data Management policies and Data Management plans

12.4% of the researchers and support staff were not sure if their institute had a Research Data Management policy. All support staff aware of the policy did know who to contact regarding the policy, however, 13.6% of the researchers did not know who they should contact. In addition, a large amount of researchers (41.4%) and support staff (21.1%) did not know if FAIR was part of the policy. For this reason, Research Data Management departments should ensure that all personnel is aware of this policy and its contents and should provide adequate training and contact options to achieve this.

### Self-efficacy and need for support

Nearly all researchers are willing to make their data FAIR, when given the right resources to do so. One of these resources includes help from experts, for the self-reported self-efficacy of researchers increases from 35.1% to 81.6% when help is offered to them. Budget and time are important as well. When researchers do have to spend their own budget, time, or both, on data FAIRification, their intention to act decreases tremendously. Researchers should, therefore, be made more aware of the fact that Research Data Management, including the FAIRification of their data, should be budgeted in a study^5^. Funders play an important role here, since they can explicitly point this out to researchers and, if necessary, allocate extra funding for them. Additionally, institutes should allocate more time and budget to FAIR Research Data Management and appoint support staff, such as data stewards and semantic data experts, that can assist researchers in the FAIRification process. We notice that support staff is increasingly recruited in institutes, however, our results also show that support staff still need training for their self-reported self-efficacy and perceived ease of use is low.

### Behavior

With an R^2^ of 0.127 the predictive power of the model regarding the behavior of researchers is low. However, the paths marked as significant in our model are relevant in practice. Researchers that believe that FAIR Data is useful for them do have a more positive attitude regarding data FAIRification. When researchers have a positive attitude towards FAIR, they are more willing to start making their data FAIR. The cross-loadings indicate that observed variables, currently associated with Awareness, Facilitating conditions, and Self-efficacy, could be removed to improve the accuracy of the model. We, however, believe that latent variables in our model can be related to one another. For example, making data FAIR by yourself (Self-efficacy) also explains if the FAIRification process is clear and understandable (Perceived ease of use). Future research should aim to determine, in depth, what steps researchers currently take to make their data FAIR, how effective they are in that (Self-efficacy), and how easy they find the steps they take (Perceived ease of use).

Since making data FAIR is still quite new to researchers, and training and tools for FAIRification still have to be developed, we believe that focusing on the researcher’s intention to act is, at this point, more meaningful than the final behavior of the researcher. The behavior of the researchers is currently rather low: only a minority of them add metadata to datasets or data elements, a minority includes FAIRification plans in a DMP, and only a small amount of researchers deposits data in a repository. In addition, an analysis of the free-text questionnaire responses, described in^25^, showed that the majority of researchers’ FAIRification efforts are focused on human readability. Only a minority is focused on achieving machine readability, for example by submitting metadata to a data repository or using standardized terminologies for describing data elements^25^. However, these metadata are essential to making research data findable and reusable for others^26^, as they allow the data owner to describe the degree to which any piece of data is available for reuse^9^.

We believe that once there are more tools, documentation, and help available that guide researchers through the FAIRification process (Structural assurance and Facilitating conditions), researchers will feel more empowered to add and publish metadata, include FAIRification plans in DMPs, and deposit data, and thus make their data more FAIR (Perceived ease of use and Self-efficacy), and will, therefore, have an increased intention to act. Once researchers are empowered and receive support to perform data FAIRification, the behavioral aspect in the model will also change.

### Strengths and limitations

This study has several strengths. First, we used an existing, validated model based on two established Technology Acceptance Model-based models. Second, we directly invited researchers that are responsible for data management, and thus FAIRification, via an EDC platform and Research Data Management departments in University Medical Centers. Lastly, we did not include any reference to FAIR into the invitation emails and popup, which made sure that potential participants that did not know about the FAIR Data Principles were also inclined to participate in the survey.

However, this study does have some limitations. The few adaptions made to the model were not validated, although the original model has been validated before. In addition, our study was carried out in The Netherlands. Although we believe our results are generalizable to other countries, there may be differences between the Dutch and other research communities. Because the FAIR Principles had their origins in the Netherlands and adherence is required by the Dutch Research Council^6^, it is reasonable to assume that the Netherlands has a higher rate of acceptance and awareness of the Principles than other countries. To comply with the General Data Protection Regulation, potential respondents were not directly contacted by the research team and were invited via a popup in an EDC platform or email by contact persons in the University Medical Centers. We are, therefore, unable to determine a response rate for our questionnaire and unable to verify the background of the respondents. Lastly, the questionnaires that were partially filled in were excluded from the analysis, which could have introduced selection bias.

### Findings in relation to other studies

Our findings are consistent with Trifan and Oliveira’s overview of the adoption and impact of the FAIR principles in the area of biomedical and life-science research^27^. Similar to our results, they found that the adoption of the FAIR Data Principles is an ongoing process within the biomedical community. A part of the factors that drive or inhibit researchers to share and reuse research data identified by Zuiderwijk *et al*.^28^ can also be observed in our SEM results: the attitude of researchers (their personal and intrinsic motivation) is the main driver for researcher’s intention to act. In addition, we found a significant difference in the interpersonal influence of researchers that have FAIR knowledge compared to researchers without this knowledge. Facilitating conditions, another factor identified by Zuiderwijk *et al*.^28^, also significantly differed between researchers with FAIR knowledge and without FAIR knowledge, which can indicate that in institutions that facilitate FAIRification, researchers are more aware of what FAIR is. However, this factor does not have a significant effect to the intention to act in our model. This can be due to the fact that making data FAIR is a relatively new concept to researchers, and that there are limited tools and help available to guide them through the FAIRification process. Lastly, our recommendation to focus more on training and tooling to make data FAIRification more accessible and comprehensible for researchers resonates with Boeckhout *et al*.’s advice to facilitate and organize data sharing using metadata standards and platforms^29^.

### Unanswered and new questions

While setting up the questionnaire, we discovered that there were no concrete self-reporting instruments available for clinical researchers that assess the effort one took to make their data FAIR. The FAIR assessment instruments that are available are rather technical, such as the FAIR metrics^30^, or generic, such as the Self-Assessment Tool to Improve the FAIRness of Your Dataset^31^. Since there was a lack of a better alternative, we, therefore, used a 4-point Likert scale in our study. We believe that each research community should develop comprehensible checklists and tooling for researchers in that community that include domain-specific information and standards.

We found a high percentage of researchers that spent at least some effort on the FAIRification of their data. However, in our experience, researchers still struggle with the FAIRification process and do not make their data FAIR at all. Future research should focus on identifying the different types of FAIRification efforts that researchers spend and determining if they actually contribute to data and metadata that is more FAIR. Lastly, since our questionnaire did not focus on a specific type of research data (e.g., genomics, imaging, or Case Report Form data), we were unable to compare FAIRification efforts and efficacy across different research contexts or data types. This comparison, however, is helpful for tailoring training, tools, and support to different groups, and future research should focus on this as well.

## Conclusion

Clinical researchers and research support staff are currently undertaking efforts to make collected data more FAIR, but the majority of them do not add human-readable and machine-readable metadata to their datasets and data elements, nor deposit their data in a repository, hampering reusability. However, a majority of the researchers are aware of the potential and usefulness of their data being FAIR for others and stated that they would ensure that their data is FAIR if they receive the right resources and help to do so. Institutions, funders, and other organizations involved in clinical research should, therefore, make training researchers and research support staff in Research Data Management and data FAIRification a priority, as well as motivating and (financially) supporting them to FAIRify and share their data. Additionally, tools should be developed that guide the researchers and support staff through the FAIRification process in a user-friendly manner.

## Supplementary information

Supplementary Table 1

Supplementary Table 2

Supplementary Table 3

Supplementary Table 4

Supplementary Table 5

Supplementary Table 6

Supplementary Table 7

## Data Availability

The questionnaire used for and the data generated or analysed during this study, with the exception of free-text questionnaire responses are available through Figshare^32^.

## Code availability

Our R scripts, used for the analysis of the data, are available on GitHub (https://github.com/martijnkersloot/perceptions_analysis).

## References

[1] van Reisen M (2020). Towards the tipping point for FAIR implementation. Data Intelligence.

[2] Wilkinson, M. D. *et al*. The FAIR guiding principles for scientific data management and stewardship. *Scientific Data* **3**, 10.1038/sdata.2016.18 (2016). 10.1038/sdata.2016.18 PMC4792175 26978244

[3] Wise J (2019). Implementation and relevance of FAIR data principles in biopharmaceutical R&D. Drug Discovery Today.

[4] Thompson M, Burger K, Kaliyaperumal R, Roos M, da Silva Santos LOB (2020). Making FAIR easy with FAIR tools: From creolization to convergence. Data Intelligence.

[5] European Commission. Horizon Europe - data management plan template. https://ec.europa.eu/info/funding-tenders/opportunities/docs/2021-2027/horizon/temp-form/report/data-management-plan-template_he_en.docx (2021).

[6] The Netherlands Organisation for Scientific Research (NWO). Data management protocol. https://www.nwo.nl/en/policies/open+science/data+management.

[7] LUMC. Research ICT. https://www.lumc.nl/research/research-ict/ (2016).

[8] Radboud University Research Data Management. FAIR principles. https://www.ru.nl/rdm/vm/fair-principles/ (2019).

[9] Mons B (2017). Cloudy, increasingly fair; revisiting the fair data guiding principles for the european open science cloud. Information Services & Use.

[10] Vesteghem C (2019). Implementing the FAIR data principles in precision oncology: review of supporting initiatives. Briefings in Bioinformatics.

[11] GO FAIR. FAIRification process. https://www.go-fair.org/fair-principles/fairification-process/ (2019).

[12] Jacobsen A (2020). A generic workflow for the data FAIRification process. Data Intelligence.

[13] Sinaci AA (2020). From raw data to FAIR data: The FAIRification workflow for health research. Methods of Information in Medicine.

[14] Jacobsen, A. *et al*. FAIR principles: Interpretations and implementation considerations, 10.1162/dint_r_00024 (2020).

[15] Joukes, E., Cornet, R., de Bruijne, M. C., de Keizer, N. F. & Abu-Hanna, A. Development and validation of a model for the adoption of structured and standardised data recording among healthcare professionals. *BMC Medical Informatics and Decision Making* **18**, 10.1186/s12911-018-0640-8 (2018). 10.1186/s12911-018-0640-8 PMC6027789 29954388

[16] Castor EDC. Castor Electronic Data Capture. https://www.castoredc.com (2020).

[17] R Core Team. *R: A Language and Environment for Statistical Computing*. R Foundation for Statistical Computing, Vienna, Austria (2020).

[18] Sarstedt, M., Ringle, C. M. & Hair, J. F. Partial least squares structural equation modeling. In *Handbook of Market Research*, 1–40, 10.1007/978-3-319-05542-8_15-1 (Springer International Publishing, 2017).

[19] Cassel C, Hackl P, Westlund AH (1999). Robustness of partial least-squares method for estimating latent variable quality structures. Journal of Applied Statistics.

[20] Sanchez, G., Trinchera, L. & Russolillo, G. *plspm: Tools for Partial Least Squares Path Modeling (PLS-PM)* (2017). R package version 0.4.9.

[21] Hair JF, Risher JJ, Sarstedt M, Ringle CM (2019). When to use and how to report the results of PLS-SEM. European Business Review.

[22] Hair Jr, J. F., Hult, G. T. M., Ringle, C. & Sarstedt, M. *A primer on partial least squares structural equation modeling (PLS-SEM)* (Sage publications, 2016).

[23] Henseler J, Ringle CM, Sarstedt M (2014). A new criterion for assessing discriminant validity in variance-based structural equation modeling. Journal of the Academy of Marketing Science.

[24] Tucker, K. *et al*. Protecting patient privacy when sharing patient-level data from clinical trials. *BMC Medical Research Methodology* **16**, 10.1186/s12874-016-0169-4 (2016). 10.1186/s12874-016-0169-4 PMC4943495 27410040

[25] Kersloot, M. G., van Damme, P., Abu-Hanna, A., Arts, D. L. & Cornet, R. *FAIRification Efforts of Clinical Researchers: The Current State of Affairs, vol. 287 of Studies in Health Technology and Informatics*, 35–39 (IOS Press, 2021). 10.3233/SHTI210807 34795075

[26] Miron, L., Gonçalves, R. S. & Musen, M. A. Obstacles to the reuse of study metadata in ClinicalTrials.gov. *Scientific Data* **7**, 10.1038/s41597-020-00780-z (2020). 10.1038/s41597-020-00780-z PMC7749162 33339830

[27] Trifan, A. & Oliveira, J. L. Towards a more reproducible biomedical research environment: Endorsement and adoption of the FAIR principles. In *Biomedical Engineering Systems and Technologies*, 453–470, 10.1007/978-3-030-46970-2_22 (Springer International Publishing, 2020).

[28] Zuiderwijk A, Shinde R, Jeng W (2020). What drives and inhibits researchers to share and use open research data? a systematic literature review to analyze factors influencing open research data adoption. PLOS ONE.

[29] Boeckhout M, Zielhuis GA, Bredenoord AL (2018). The FAIR guiding principles for data stewardship: fair enough?. European Journal of Human Genetics.

[30] Wilkinson, M. D. *et al*. A design framework and exemplar metrics for FAIRness. *Scientific Data* **5**, 10.1038/sdata.2018.118 (2018). 10.1038/sdata.2018.118 PMC6018520 29944145

[31] Data Archiving and Networked Services. Self-assessment tool to improve the FAIRness of your dataset. https://satifyd.dans.knaw.nl/ (2019).

[32] Kersloot MG, Abu-Hanna A, Cornet R, Arts DL (2021). figshare.

